# An Overview of Physiologically-Based Pharmacokinetic Models for Forensic Science

**DOI:** 10.3390/toxics11020126

**Published:** 2023-01-28

**Authors:** Kiara Fairman, Me-Kyoung Choi, Pavani Gonnabathula, Annie Lumen, Andrew Worth, Alicia Paini, Miao Li

**Affiliations:** 1 Division of Biochemical Toxicology, National Center for Toxicological Research, United States Food and Drug Administration, Jefferson, AR 72079, USA; 2 European Commission, Joint Research Centre (JRC), 21027 Ispra, Italy; 3 esqLABS GmbH, 26683 Saterland, Germany

**Keywords:** PBPK model, post-mortem redistribution, exposure, illicit drug, substance of abuse

## Abstract

A physiologically-based pharmacokinetic (PBPK) model represents the structural components of the body with physiologically relevant compartments connected via blood flow rates described by mathematical equations to determine drug disposition. PBPK models are used in the pharmaceutical sector for drug development, precision medicine, and the chemical industry to predict safe levels of exposure during the registration of chemical substances. However, one area of application where PBPK models have been scarcely used is forensic science. In this review, we give an overview of PBPK models successfully developed for several illicit drugs and environmental chemicals that could be applied for forensic interpretation, highlighting the gaps, uncertainties, and limitations.

## 1. Introduction

**Figure 1:**
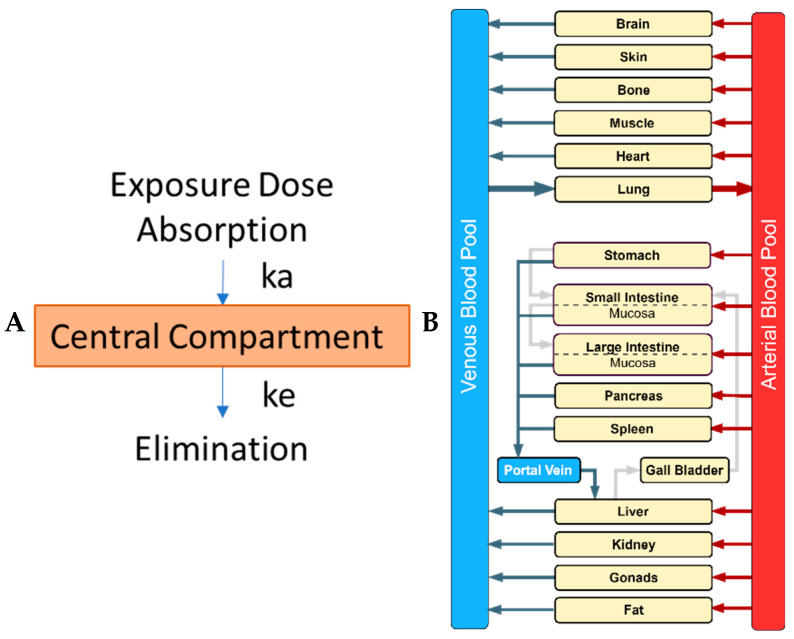
Comparison of a classical empirical compartmental model and a mechanistic physiologically-based pharmacokinetic (PBPK) model. (**A**) In the classical one-compartment model, a drug/chemical enters a central compartment, representing all tissues, by absorption, and is governed by the absorption rate constant (ka) while its elimination is described by the elimination rate constant (ke) (picture made using Microsoft ppt); (**B**) In the whole-body PBPK model, major organs/tissues are represented by compartments (boxes), connected by blood flows (venous and arterial blood pool; blue and red box, respectively), and flow is represented by the arrows (picture made using drawio.io).

A physiologically-based pharmacokinetic (PBPK) model represents the mathematical description of the body by means of differential equations interposed with physiologically accurate organ compartments as a structural representation of the body. Each organ compartment is connected by blood flows represented by differential equations that subsequently coalesce to yield predictions of drug or toxicant pharmacokinetics. These mathematical models can span from a simple empirical one-compartmental model to a more refined and complex PBPK model, which could include all organs (Figure 1). PBPK models describe the absorption, distribution, biotransformation (metabolism), and elimination (so-called ADME processes) of a xenobiotic from the body [1]. Exposure or uptake of chemicals/drugs is also described within the model as oral, dermal, subcutaneous, inhalation, or direct injection into the blood (intravenous). Each parameter in the model represents a physiological, physicochemical, or biochemical value that influences the ADME processes [1]. Population variability and uncertainty can be described by assigning distributions to each model parameter [2,3,4]. PBPK models can describe healthy individuals as well as susceptible populations (e.g., pediatric, elderly, pregnant, and immunocompromised). A recent systematic review and curation of available PBPK models in the literature was published by Thompson et al. (2021) [5]. From this review, model information, including species, sex, life-stage, route of administration, software platform used, and the availability of model equations, was captured for 7541 PBPK models and for 1150 unique chemicals associated with these models [5].

PBPK models have emerged as a tool spanning many disciplines. They are used in the pharmaceutical sector for drug development and precision dosing in medicine [6], as well as in the chemical industry to register chemical substances. They are also applied by agencies (EMA, EFSA, US EPA, and US FDA among others) to estimate safe exposure limits of drugs and chemicals (drug and chemical risk assessment) for humans (e.g., workers, consumers, and patients) [7]. Recently, such models have also been applied to veterinary medicine to describe chemical/drug intake and distribution in farm animals [8,9,10,11,12]. While in the context of environmental risk assessment, PBPK models have been developed for a range of wild species [13,14,15,16,17,18]. One area of application where PBPK models have been used infrequently, however, is in forensic science. The US Department of Justice defines forensic science as “a critical element of the justice system” that applies science to analyze evidence from crime scenes for emerging findings during a criminal investigation [19]. Part of forensic science is the interpretation of laboratory results by forensic toxicologists. Such interpretations may be used to prove acute or chronic use of a substance, to determine the cause of death, or to establish the timeframe in which a crime was committed under the influence of a substance [20]. Forensic application is a challenging task because it can be difficult to link and extrapolate between biological evidence (e.g., effect–damage) and a numerical value (e.g., drug concentration in plasma, tissue, etc.) [20]. Interpretation of post-mortem forensic data to determine a cause of death can be challenging due to post-mortem redistribution (PMR) phenomena. PBPK models can be applied to establish concentration-time profiles of the drug(s) in blood and different tissues in humans to resolve such limitations. A PBPK model allows the computation and prediction of the concentrations of a chemical (and its metabolites) within the body over time from a given exposure. This can be measured as Cmax (maximum concentration) for acute (accidental) exposure, or as the area under (the concentration-time) curve (AUC) for chronic/long exposure.

In this review, we provide an overview of PBPK models successfully developed for different drugs and environmental chemicals with different exposure scenarios that could be applied for forensic interpretation, highlighting the gaps, uncertainties, and limitations.

## 2. Search Strategy

**Figure 2:**
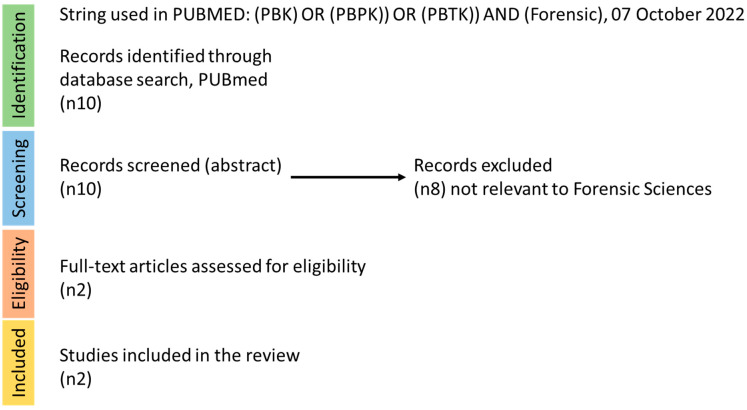
Literature search strategy for forensic science and PBPK models. The steps of identification, screening, eligibility, and inclusion are depicted in this figure, with the relevant recorded hits per step.

When searching the literature (using the following string: (((PBK) OR (PBPK)) OR (PBTK)) AND (Forensic), in PUBMED, on 22 July 2020) 10 hits were recorded. However, based on the abstract screening, only two papers appeared to contain PBPK models used explicitly for forensic purposes (Figure 2). Bravo-Gómez and co-workers applied PBPK modelling as a tool for the forensic interpretation of cocaine [8] ADME characteristics, while Schaefer and colleagues performed a similar analysis for morphine [9]. Therefore, while discussing these papers briefly, we will also detail some instances in which PBPK models are applied for substances in short and long-term exposure scenarios that may be useful in forensic investigations.

## 3. PBPK Model Development and Resources

As PBPK models are quite new in the field of forensic toxicology, we will present a short description of how these models can be developed. In order to build a PBPK model, a six-steps approach can be followed. These steps are described in several international guidance documents [21,22,23,24,25,26] and are briefly outlined below:

Step 1. Problem formulation: The purpose of why the model is built should be determined.
Step 2. Model conceptualization: The structure of the PBPK model should be defined and informed by the problem formulation, knowledge of the underlying physiological and biokinetic mechanisms, and the availability of suitable data. The schematic model structure should be translated to mathematical equations to be implemented computationally.
Step 3. Model parameterization: PBPK models are built using three sets of parameters: (i) physiological and anatomical parameters, with representative reference parameters obtained from the species under study (animal or human); (ii) biokinetic/ADME properties, such as clearance, which can be acquired using in vitro methods or by fitting the model to an in vivo data set; and (iii) physico-chemical parameters, such as lipophilicity, which are experimentally derived or obtained using in silico approaches such as quantitative structure activity relationship (QSAR) models. Several resources for model parameterization have been mapped [27].
Step 4. Computer (software) implementation: This includes the choice of programming language to translate mathematical equations to computer code and the solver for execution of the model code. Currently, several open access and open-source modelling platforms, such as IndusChemFAte (Cefic LRI, http://cefic-lri.org/toolbox/induschemfate/), High-Throughput Toxicokinetics (httk)-r package (https://cran.r-project.org/web/packages/httk/index.html), MEGEN-RVis (https://megen.useconnect.co.uk/), PLETHEM (http://www.scitovation.com/plethem.html), MERLIN-EXPO (https://merlin-expo.eu/), and PK-Sim (www.systems-biology.com), and license-based platforms such as GastroPlus (www.simulations-plus.com) and SimCyp (https://www.certara.com), are available to individuals possessing varying degrees of expertise in PBPK modeling. These platforms provide different computational tools that allow non-programmers to develop and run model simulations with varying options to gain a better understanding and interpretation of model outputs. However, programmers or users with modeling skills can also use R, MATLAB, and Berkeley Madonna software to develop customized PBPK models.
Step 5. Model performance: Validation of model prediction against actual observed clinical data—in vivo data [21,22].
Step 6. Report and disseminate the model and simulations in a transparent and a “FAIR” way [28,29]. FAIR represents the Findability, Accessibility, Interoperability, and Reusability of scientific data. The FAIR guiding principles were first formally published in 2016 by Wilkinson et al. (2016) [30]. PBPK models can be reported using established templates [31] and or an international guidance document [21,22].

## 4. PBPK Model Applications in Forensic Science

As PBPK models have different applications in several fields, they could also be applied in forensic science to help toxicologists interpret forensic evidence. The interpretation of the data and association with a response effect starts from the actual exposure. We cover both acute and non-acute exposure scenarios (e.g., short, long, and chronic exposure) for both drugs and environmental chemicals because these potential scenarios are where PBPK models could be informative. For each of these exposure scenarios, some illustrative examples based on available literature are presented. Since only two articles were available specifically for forensics, we cover non-forensic PBPK models that could be used for some commonly encountered substances in overdose or other forensic exposure scenarios. These models could be adapted for use in forensic science with additional considerations such as postmortem redistribution, which is discussed in detail later (Section 7 and Section 8).

## 5. Illegally Used Drugs

In 2019, at least 49% of all Americans used prescription drugs [32] and at least 13% used illicit drugs [33]. With such a large portion of the population taking some type of drug, there is ample possibility for adverse events to occur in both acute or long-term dosing scenarios due to user, prescriber, or distributor negligence. Depending on the available data, PBPK models can simulate various dosing exposure types over extended time frames such as months and years, or for just a few hours or less.

The following section considers various types of exposures to drugs and alcohol via PBPK modeling and how these techniques could be used in forensic toxicology. We initially consider PBPK models for prescription drugs used inappropriately and illicit drugs, including the two models specifically developed for forensic use looking at morphine and cocaine as mentioned above (full list available in Supplementary Material, Table S1). Then, we describe PBPK models of other commonly abused drugs that have the potential to be used for forensic science purposes.

### 5.1. Acute, Short-Term, & Long-Term Exposure

Acute exposure is not as well defined in humans as it is in animals, which creates some difficulty in classifying existing PBPK models for forensic use. In humans, acute exposure is synonymous with short exposure and can be the result of a single exposure incidence or a short-term one, while chronic acute exposure usually lasts less than a few weeks [34]. Acute exposure can immediately cause detrimental effects if the threshold of toxicity is exceeded at the target site. Detrimental effects are dependent upon the chemical or drug and can occur after a single dose or through repeated exposure causing accumulation via long half-lives or irreversible binding. PBPK models can be especially helpful when determining the target site concentration since the compartments are physiologically-based representations. This allows for the estimation of time-dependent organ concentrations with partition coefficients, even when only plasma concentrations are available. Organ concentrations, in relation to time of dose or ingestions, are useful tools in forensic science for recreating the scene of a criminal event.

Acute exposures are often caused by accident or intentional poisonings of oneself or others. Common causes of acute exposure are dosing errors and illicit drugs overdoses. Furthermore, illicit drugs are not regulated and can even be contaminated with other substances. Thus, we discuss both single and multiple drug exposures of these substances with PBPK.

Long-term exposure is composed of sub-chronic to chronic exposure lasting from a few weeks to years [34]. Forensic PBPK models could be used to estimate short and long-term exposures because these models are able to estimate concentrations for single dose and multiple dose scenarios. Therefore, steady state concentrations can be reached and predicted for the various organs that have been parameterized. This contrasts with classical pharmacokinetics (PK) since classical PK is not organ specific and does not allow individual compartment concentrations of substances to be estimated. Although illicit drugs can be used over long time frames, overdoses and, thus, the forensic implication may be more prevalent for the acute setting. Examples of long-term drug or alcohol use simulated with PBPK model scenarios were not found in our literature search. Even still, long-term physiological effects could further complicate the pharmacokinetics and alter parameters. For example, long-term opioid use can result in peristalsis and the slow transit of drugs through the gastrointestinal tract. Amphetamines and stimulants could increase metabolism and cause decreased drug half-life. Chronic alcohol use can induce CYP2E1 and increase alcohol metabolism, as well as cause pharmacodynamic (PD) effects such as Wernicke’s encephalitis. Therefore, if long-term PBPK models for illicit drugs or alcohol are created for use in forensic science, altered physiological parameters must be considered.

#### 5.1.1. Opioids

In the United States 10.1 million people misused and 70,360 people died from opioids in 2019 [35]. The sheer number of people affected caused the US Department of Health and Human Services to declare the opioid crisis a public health emergency in 2017 [35]. Although only a few examples of PBPK model use for forensic science purposes have been reported, one such example resides in the opioid domain. Additional models exist for opioid drugs; however, these are not explicitly for forensic purposes, albeit they could be repurposed for such, depending on the desired questions that the model is set to address.

One of the few examples of PBPK modeling use in forensics was with a suspected fatal morphine dose in a 98 year old man. In this example, the subject was in palliative care at a nursing home, suffered a fall, and was subsequently hospitalized, where the accusation of malfeasant dosing prompted the creation of a PBPK model to investigate possible dose scenarios. A PBPK model was used to estimate internal tissues concentrations that would support morphine overdosing or the associated reported clinical dose. The PBPK model of morphine and its morphine 6-glucoronide metabolite was calibrated and validated with clinical data; then samples from the patient’s blood, brain, liver, and lung were obtained. Various conditions, such as anuresis before death, hepatic failure, and postmortem redistributions due to cell lysis, were considered. The PBPK model did not support an opioid overdose due to the many uncertainties that existed, such as possible renal and hepatic failure, which could also cause tissue concentrations to mimic a high dose. The autopsy and forensic toxicology suggested that amongst other things a high, toxic concentration of morphine may have attributed to the subject’s death [36].

Other oral, IV, or subcutaneous opioid PBPK models exist for morphine [37,38,39,40,41,42,43,44,45,46,47,48], alfentanil [49,50,51,52], remifentanil [53,54,55], sufentanil [56], tramadol [56,57,58,59,60,61], fentanyl [62,63,64,65,66,67], oxycodone [40,64,68,69], buprenorphine [64,70,71,72,73,74,75], codeine [76,77], methadone [78,79,80,81,82,83,84], and carfentanil to predict drug-drug interactions and drug disposition in the plasma and various organs. However, carfentanil is one of the few opioids that mention the possible application of PBPK modeling in forensic science. Carfentanil is an illegal, highly potent synthetic opioid, dosed in picograms, that poses a threat to both public health and chemical weapons defense. A carfentanil PBPK model mentioning forensic applications was detailed in the 2018 research of Feasel et al. [85]. The PBPK model was validated successfully using rabbit physiology, then extrapolated to humans, and optimized for dose-equivalence. This model could be applied, for instance, in a case study by Canneart et al. 2018 [86], where a 21 year old male may have consumed up to 50 mg of a carfentanil laced substance labeled “C.50” in an apparent suicide. A forensic toxicologist roughly estimated the ingested amount using samples collected at autopsy; however, a PBPK model could give a better estimated range of the dose using both plasma and tissue concentration.

#### 5.1.2. Psychostimulants

Stimulant drugs, such as amphetamines and cocaine, can be prescription drugs or illicit drugs. With the wide range of applications for disease and recreational use, in addition to the addictive nature of these substances, there has been a surge in recent years in the deaths related to this class of drugs [33]. Bravo et al. created a diffusion-limited PBPK model for cocaine using PK data from animal studies and extrapolated it to humans to predict blood and tissue concentrations [20]. Literature reported pharmacodynamic (e.g., psychoactive effects) time course outcomes were compared with the simulated PK data and found to match tissue-specific symptoms [20]. The authors specifically recommended this as a tool for forensic investigation and utilized the model to compare tissue distribution [20]. In this situation, pharmacodynamic endpoints interwoven with interspecies blood plasma concentration were accurately extrapolated to predicted human concentration-time courses. This model type could be a tool in determining the possible time of cocaine exposure in a criminal case [33].

#### 5.1.3. Psychedelics

Psychedelic drugs have been around for centuries and are often a part of ancient rituals around the world [87]. Although most psychedelics are illegal and therefore classified as illicit drugs, some more progressive research findings suggest some usefulness of these drugs for the treatment of a variety of disease indications. Furthermore, since these drugs tend to act upon serotonergic receptors as full or partial agonists to 5-hydroxytryptamine (5-HT) 2A receptors, there may be potential for drug-drug interactions with anti-depressants, as well as some off-target effects on other 5-HT receptors substrates, especially considering off-target effects due to reduced selectivity at high doses [87,88].

##### Psilocybin and Psilocin

Psilocybin and psilocin are the major psychoactive substances found in magic mushrooms (Psilocybe cubensis) [89]. The body rapidly transforms psilocybin into psilocin, which exerts a psychedelic effect by activating the 5-HT2A receptor in the brain [89]. Because of this psychedelic effect, magic mushrooms can cause euphoria, hallucinations (mental and auditory), and perception changes. Psilocybin has been studied as a treatment for a variety of psychological disorders including depression, suicide attempts, obsessive-compulsive disorder, alcohol-use disorder, tobacco-use disorder, and resistant depression [89].

Musikaphongsakul et al. developed a psilocin PBPK model that described concentration-time profiles in plasma from both rats and humans [89]. Furthermore, after intravenous or oral dosing of psilocybin, the PBPK model predicted psilocin concentration-time profiles in other tissues, such as the brain, which is the primary target organ of the psychoactive compound in magic mushrooms. Although the model was originally proposed to help with a safer psilocybin dosage regimen for patients with relevant disease conditions, it could also be used to identify instances of malfeasant dosing in forensic cases.

##### Mitragynine

Mitragynine is a naturally occurring indole alkaloid that can be isolated from kratom leaves (Mitragyna speciosa Korth) [90]. To understand the drug’s distribution in organs, a physiologically-based pharmacokinetic (PBPK) model was developed to predict mitragynine concentrations in plasma and organs of interest in rats and humans [90]. To determine safer mitragynine dosing regimens, this model can be used to predict steady state mitragynine concentrations. It may be effective in predicting mitragynine levels in kratom overdose patients. This model could predict the blood and brain concentration-time profiles of mitragynine in rats and humans under various dosing scenarios and could serve as a guide to establish safer dosing guidelines.

#### 5.1.4. Multidrug Combinations

The CDC has reported that multidrug combinations can cause an increased risk of death and overdose, especially when opioids are mixed with psychostimulants [33]. The presence of PBPK models for cocaine and the opioid drug class being used explicitly in the forensic setting is fitting as the combination of these two drugs is the cause of many overdose deaths in the US [33]. Having the tools to recreate possible exposures of each individual drug and even concomitant exposures create a more robust picture of what may have occurred prior to death or a criminal incident. In a recent study by Cheng and co-workers, a PBPK model was applied to simulate drug-drug interactions for two drugs (fentanyl and cocaine) to predict their ADME profile [90]. This was performed to understand the synergism the two drugs have on the human body since drug overdoses caused by fentanyl-laced cocaine are on the rise [90]. Due to drug synergy and an increase in side effects, polydrug addiction can cause more risk than addiction to a single drug. For example, drug and alcohol use often occur simultaneously, with 74% of adults with substance-use disorders also having alcohol-use disorders [91]. Thus, modeling of polypharmacy in the forensic setting can be further complicated due to the potential drug-drug interactions (DDI) that often occur with alcohol use. However, PBPK modeling is particularly useful as a modeling and simulation tool for predicting pharmacokinetics of drugs or drug combinations based on mechanistic processes, such as tandem enzymes inhibition, induction, or competitive inhibition, which could result in drug concentrations increasing or decreasing in the plasma or target organ tissues. For example, while cocaine and many opioids, such as hydrocodone, do not share enzymes in their major elimination pathways, each are serotonergic drugs and concomitant use could increase neural serotonin levels causing serotonin syndrome which could be life threatening [92]. Yet, there are also reports that chronic cocaine use may cause the induction of CYP3A4 and reduce the levels of some opioids [93]. PBPK models can be used to predict scenarios of possible DDIs in specific tissues (e.g., blood, brain, and liver, etc.) that could potentially pose a risk to human health.

#### 5.1.5. Alcohol

The CDC estimates that more than 140,000 people die of excessive alcohol use each year in the US [94]. Alcohol exposure can occur through various routes, but is typically through ingestion, inhalation, and dermal absorption. Social drinking and abuse can result in accidental, acute, and long-term exposure, ultimately leading to complications such as liver injury. With the prevalence and access to alcohol in nearly every society, it is not surprising that there are many PBPK models for ethanol. However, no published articles specifically addressing PBPK models for ethanol in forensic investigations were found. Therefore, the following section is an overview of the available PBPK models for ethanol simulating exposure types, biological variability, and mechanistic processes.

The most relevant PBPK models for alcohol that could be utilized for forensic purposes can simulate exposure through acute and repeat exposure modes. For example, PBPK models for acute exposures have been utilized for occupational exposures and accidental and intentional environmental exposures. Ethanol exposure as an occupational hazard, through either accidental inhalation or dermal exposure from hand sanitizers, has been explored by multiple research groups through various PBPK models [95,96,97,98]. Environmental exposure to alcohol from a variety of sources such as fuel or non-occupation hand sanitizer use has been published by multiple groups as well [99,100,101,102]. Intentional exposure, either through common social consumption routes or healthy volunteer research cohorts, were also modeled by multiple groups [103,104]. Considering that ethanol is a chemical solvent and used in some hand sanitizers, occupational exposures and predicted drug concentrations in the organ of interest could be utilized in a court of law to determine overexposure. Consequently, this could then be used to determine employer negligence or an unsafe work environment.

Additionally, genetic variation and inter- and intraindividual considerations have been considered in PBPK modeling of ethanol than could aid in PBPK modeling’s use in forensic investigations. Many PBPK models exist for improving and investigating breath alcohol concentration estimations based on inter- and intravariability in animals and humans [105,106,107,108,109,110,111]. Neurotoxic risk assessment using PBPK models to determine the acute effects of ethanol in rats is also covered in the literature [112]. Specific enzyme polymorphisms in acetaldehyde dehydrogenase (ALDH) [113], alcohol dehydrogenase (ALD) [113,114,115,116,117], and CYP2E1 [118,119] have also been addressed for ethanol metabolism to simulate individual genomics, population simulations, and interindividual, and interracial variability. Although interracial variability is not defined in the Loizou et al. 2004 article, interpopulation may be a more appropriate term. [113,114,116,117,118,119,120]. Accounting for variations in the PBPK model allows for more accurate prediction of PK outcomes within a population or for an individual if their specific genetic profile is known or postulated. For instance, if a poor metabolizer with low ALDH activity is simulated, a higher-than-normal blood alcohol level would be expected and could be predicted based on the amount of alcohol ingested. Conversely, one could estimate the amount ingested based on an internal organ concentration or breath alcohol level using reverse dosimetry techniques with PBPK modeling.

Mechanistic examples of PBPK used for ethanol further highlight the utility of these models in reverse dosimetry, diverse populations, and cross species extrapolation. Sadighi et al. 2021 [121] used PBPK modeling to simulate the time concentration of ethanol in various organs, particularly paying attention to Cmax, time to maximum concentration (Tmax), and AUC to determine the quickest, highest exposure. The PBPK model was also used to predict which organ had the greatest exposure overtime and guided the design of in vitro experiments for organ damage from alcohol or drug interactions [121]. Kirman et al. [122] compared allometric scaling and PBPK modeling based on low and high doses of ethanol and other chemicals. Levitt and colleagues also addressed more mechanistic aspects of PBPK modeling [123] and determined that gastric metabolism was insignificant, but that ethanol did exhibit a food effect [124]. They found that antecubital vein concentration was sufficient for modeling whole body PBPK in ethanol [125]. Additional mechanistic studies with ethanol were performed by Liu et al. in 2019 [126] where they used PBPK modeling to compare ethanol penetration through the blood brain barrier using a brain microphysiological system and in vivo data to further support their microphysiological model. Morzorati et al. 2002 [127] presented a complementary study to Liu’s study by using PBPK modeling of rats to help achieve target arterial alcohol concentrations. In experiments where fluctuating alcohol levels may cause problems with the study results, Morzorati’s technique of using arterial alcohol concentrations was more indicative of a person’s impaired state [127]. These mechanistic studies could help in the experimental design or evaluation of how data is collected, thus providing a better perspective of potential errors in clinical or criminal justice practice.

## 6. Environmental Chemicals

Chemical exposure defines how much and how often one may be exposed to chemicals. The degree of chemical exposure and the amount of chemical found in the blood and organs at various times are compared using a PBPK model. This method aids in determining if a dangerous level of a chemical would be discovered in a person’s blood or an organ after exposure to a certain amount of a chemical. PBPK modeling could help forensic scientists to investigate environmental crimes and contamination events.

### 6.1. Acute, Short-Term, and Accidental Environmental Chemical Exposure

In the area of environmental toxicology, chemical concentrations in different tissues offer a unique way to monitor human exposure to different chemicals. Especially for the persistent organic pollutants, as they have longer half-lives, concentrations from biopsy samples could provide valuable data for environmental and toxicological modelling. Based on our current technology, there is no widely accepted way to monitor chemical concentrations in human tissues without invasive methods.

Another branch of forensic science, “industrial forensics,” can benefit from PBPK modelling simulations as well. Industrial forensics is a holistic-based analytical and consultative response to problems encountered at any stage in the industrial manufacturing process [128]. If we look back in time, an example is the “melamine crisis” that hit Asia in 2007–2008, where infant formula was spiked with melamine to increase the protein content (adulteration of food product, fraud). This led to kidney failure and, as a consequence, several fatalities in infants. PBPK models could be used to understand the fate and distribution of chemicals at different life stages, allowing for a rapid understanding of a chemical’s exposure, mode of action, and appropriate remediations [129].

### 6.2. Long-Term Environmental Chemical Exposure

#### 6.2.1. Cyanide and Human Continuous Cyanide Inhalation Predictor (HCCIP)

Hydrogen cyanide (HCN) is a very hazardous gas that has both acute and long-term effects. Human exposure results from natural or industrial processes that release gaseous HCN into the atmosphere. Existing PBPK models cannot clearly simulate continuous HCN inhalation or predict HCN levels in inhaled air. Therefore, a Human Continuous Cyanide Inhalation Predictor (HCCIP) was developed utilizing extensive data from the PBPK model on cyanide ingestion. HCCIP is composed of the lungs, kidneys, liver, and slowly perfused tissue. HCCIP can predict cyanide concentration-time courses in both the human body and exhaled air [130]. The model was validated when the simulation results matched the datasets. The HCCIP model is a useful tool for determining the risk of long-term HCN inhalation [130].

Forensic data are also commonly used for assessing the long-term exposure to environmental chemicals, especially for persistent organic pollutants (POPs). POPs are man-made chemicals which are persistent in the environment and bioaccumulate through the food chain [131], including dichlorodiphenyltrichloroethane (DDT), polychlorinated biphenyls (PCBs), polyaromatic hydrocarbons (PAHs), polybrominated diphenyl ethers (PBDEs), and perfluoroalkyl substances (PFAS) [132]. Generally, biomonitoring studies only report the serum or urine concentrations after exposure to environmental chemicals. Post-mortem data are important additions to biomonitoring data to better understand the tissue distribution of POPs. Post-mortem data are available for legacy POPs, including polychlorinated biphenyls (PCBs) and polybrominated diphenyl ethers (PBDEs), in addition to emerging POPs such as perfluorooctane sulfonate (PFOS) and perfluorooctanoic acid (PFOA).

#### 6.2.2. Perfluoroalkyl and Polyfluoroalkyl Substances (PFAS)

PFAS chemicals, known as the forever chemicals, are the emerging POPs and with rising concern for risk to human health. PFAS have been considered as a “worldwide public health threat” and several class action lawsuits have been filed in the past 20 years, as highlighted in the movie Dark Waters.

The development of PBPK models for PFAS is important to better understand the distribution of PFAS in humans. The first several PBPK models for PFAS were based on animal pharmacokinetic data and then extrapolated to humans [133,134]. However, due to the interspecies differences, the human PBPK models extrapolated from animal data still have a lot of uncertainties [135], especially large interspecies differences exist in PFAS toxicokinetics. A study in 2013 [136] has measured the concentrations of 21 perfluoroalkyl substances in five different tissues from human autopsy samples of 20 individuals from Catalonia in Spain. Even though post-mortem redistribution was not considered and there was large interindividual variability in this study, it is still the most comprehensive information available for PFAS distribution in humans. Several PBPK models [135,137,138] for PFAS have used the tissue concentrations data from this study to predict model parameters or validate model prediction. Tissue concentrations from post-mortem reports are key information to validate PBPK model performance, especially when dealing with chemicals with significant interspecies differences and animal tissue data cannot well represent the pharmacokinetic in humans.

#### 6.2.3. Trichloroethylene (TCE)

Trichloroethylene (TCE) is a halocarbon commonly used as an industrial solvent. Because of its pleasant psychotropic effect, which is linked to sniffing it, trichloroethylene misuse causes abrupt mortality [139]. Repeated inhalation has the potential to result in a dangerous and unregulated systemic accumulation of trichloroethylene, which could then result in central nervous system depression, unconsciousness, and fatal cardiorespiratory arrest [139].

A PBPK model for TCE and its major metabolite, trichloroacetic acid (TCA), was developed and optimized for humans [140]. The optimized human PBPK model provides an excellent description of TCE and TCA kinetics [140]. The predictions were accurate for TCA plasma concentrations after repeated TCE inhalation, an accidental exposure scenario that is common in the workplace [140]. The human TCE PBPK model can be used to estimate dose metrics resulting from TCE exposures [140].

## 7. PBPK Modelling in Post-Mortem Investigations

### 7.1. Considerations of Organ-Specific Toxicity (Cardiotoxicity and Drug Induced Liver Injury) in Forensic Cases

PBPK modelling has the advantage of predicting the chemical or drug concentrations at the organ level. The organ concentrations predicted by PBPK models can help to further explore the toxicity mechanism. However, it is not possible to obtain organ or tissue concentrations of drugs or chemicals from clinical trials or biomonitoring. The tissue concentrations from post-mortem reports can help to increase confidence by using predicted tissue concentrations from PBPK models. The cardiotoxicity of doxorubicin is one of the toxicities limiting the use of doxorubicin for cancer patients. However, the mechanism of the cardiotoxicity caused by doxorubicin remains incompletely understood. PBPK modelling can help the dynamic changes of doxorubicin concentrations in the heart. A recent PBPK model for doxorubicin in humans [141] has used post-mortem data [142] to validate PBPK model performance on tissue concentrations, including heart concentrations. In addition, drug induced liver injury (DILI), which is liver injury caused by medicines and herbal, or dietary supplements, is also one of the major organ specific toxicities. DILI can result in both acute or chronic liver disease after short or long-term exposure. DILI can occur in clinical trials or during post-marketing monitoring. A liver autopsy is a way to identify the causality of liver injury post-mortem [143]. Drug concentrations from post-mortem examinations (Table 1) could provide the data necessary to determine drug distribution in different organs. Even though clinical trials provide information on drug safety and efficacy, post-market monitoring ensures drug safety and can prevent potential adverse effects in the general population over time. Forensic science could provide additional safety information for approved drugs in the event of inappropriate use, improving post-marketing drug monitoring (for example, amiodarone and tacrolimus biopsy and autopsy).

### 7.2. Post-Mortem Considerations

The development of a PBPK model is an iterative learning, confirming, and refining approach. In the continuous assessment for improving model predictions and involving mechanistic-based equations, the effects of PMR should be considered while applying data from post-mortem examinations to develop PBPK models or improve the existing PBPK models [144].

Post-mortem examinations provide information about drug-related deaths and possibly drug toxicity. Some of the preferred specimens collected could vary by case, but at least more than one blood specimen and urine and urine are typically collected. Table 1 lists some of the prevalent drugs and chemicals studied in the forensic science field using tissue samples from biopsies and post-mortem autopsies and could be applied when building or improving PBPK models. For example, to validate ongoing brain models for PBPK modelling, modelers can only use cerebrospinal fluid concentrations to verify model performance. The brain tissue concentrations from forensic reports are the only direct concentration for humans in vivo; applying those data in PBPK modeling would increase our confidence for model predictions. Blood is the most crucial sample to collect because substances found in it may be closely tied to a physiological or pharmacological effect and indicate recent drug use or chemical exposure. When interpreting the sample results, it is essential to consider the redistribution. The PMR, which refers to changes in the concentrations of drug after death but prior to autopsy, needs to be considered when using post-mortem blood or tissue concentrations to calibrate model parameters or validate model performance when developing a PBPK model. The complex processes of drug reservoir diffusion, cell lysis, and putrefaction, as well as the specific pharmacokinetic properties of the drugs, may all contribute to the changes [145]. This is particularly concerning, as many toxic chemicals and drugs are lipophilic weak bases, have a large volume of distribution, and will undergo redistribution after death, influenced by their physiochemical properties and sampling site. Papers [145,146] have summarized different factors that contribute to PMR, mentioning the computational models which may help to predict the effects of PMR for different drugs. Papers [147,148,149] have been published to model the PMR for drugs with diverse structures. The femoral blood is least affected by redistribution after death, but tissue-bound drugs can still diffuse from higher concentrations in organs into the blood, thereby increasing the concentrations [150]. Consequently, samples may not necessarily reflect the concentrations at the time of death. Not considering the impact of PMR on post-mortem blood concentrations skews the pharmacokinetic interpretation of different drugs, compounding the differences due to sampling sites in a time-dependent manner [151].

### 7.3. PBPK Model Gaps, Uncertainties, and Future Needs

In the initial literature search, very few articles were found addressing PBPK models for forensic applications (using the keywords selected). However, when looking at the different drug and chemical classes, PBPK models developed for substances on a case-by-case basis to assess their fate and distribution were found for: opioids, amphetamines, “magic” mushrooms, serotonergic psychedelics, psychoactive drugs (alcohol), and environmental chemicals (POPs, PFAS, and melamine, among others). Although drug PBPK models were available, comparisons to biopsy and post-mortem data would be useful to determine their utility as a forensic tool in drug concentration prediction and building dose-exposure relationships.

PMR makes interpretation of concentrations obtained from post-mortem samples difficult because the interactions between drugs and tissues change after death. Some studies show that PMR may not correlate strongly with physiochemical drug properties. Nonetheless, the site, time, and sample condition are responsible for variation in post-mortem concentrations [152]. Due to the uncertainties between the time of death and when blood concentrations are measured, post-mortem measurements should be used carefully. In addition, continued efforts to establish PMR concentration reference values are needed.

Polypharmacy is often a factor in drug overdose and death; however, the publications utilizing PBPK models to assess DDI in a forensic setting are lacking. As previously reported, the only example of a forensic PBPK model for a prescription drug was a potential morphine overdose. Although this area is sparse, one could still gather the appropriate information needed from existing published models and have much of the desired information to begin building a PBPK model specifically for forensic applications. Inhibition and induction values would also need to be collected or generated by experimentation to make reasonable predictions for long-term exposure scenarios. Again, plasma, biopsy, or post-mortem data, where available, may still be needed to validate the model.

Recently, a paper describing the current ongoing debate in Italy on substances of abuse, mainly directed at understanding and treating addictions, was published [153]. The paper clearly stated that non-animal alternatives, such as PBPK models, are encouraged in the field of forensics and in the area of xenotransplants and substances of abuse [153]. This further emphasizes the growing use and acceptance of PBPK models, as well as the need to expand their use in forensic science.

## 8. Discussion and Conclusions

**Figure 3:**
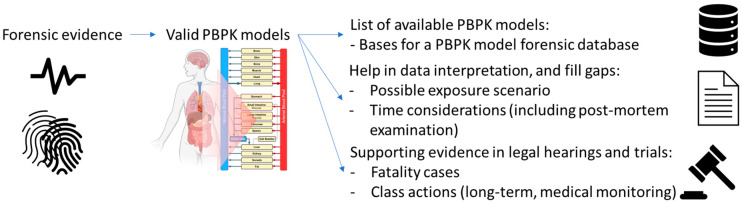
Needs and potential for PBPK models in forensic science. A valid PBPK model can be used in interpretation of data and to fill gaps, providing supporting evidence in hearings and trials. The valid PBPK model could subsequentially be included in a PBPK model database, which would be valuable for other investigations.

PBPK models have different applications in several fields and can also be applied in forensic science to support the interpretation of data. The interpretation of the data associated with an effect starts from the actual exposure for both drug abuse and environmental chemical accidents. For each of these exposure scenarios, examples were given. PBPK modelling can play a role in evaluating short- and long-term exposure scenarios, as this method could help reconstruct the exposure during an investigation, identifying exposure time, dose, and other patterns by estimating the amount of drug or chemical accumulated in specific organ tissues. Thus, when an organ biopsy or autopsy is performed and the concentration of a specific drug or chemical in the sample is measured, reverse dosimetry (understanding from a concentration in the organ how much dose an individual should have been exposed to using PBPK model) can be performed to determine the potential length or quantity of exposure. This would be most applicable in poorly perfused tissues, such as adipose and bone, where elimination is slow and allows for significant accumulation over time. Furthermore, the ability of PBPK modelling to simulate long-term drug or chemical exposure-based enzyme induction or inhibition at a certain time or exposure point could provide more accurate simulation results [154]. Therefore, when a fatal exposure is being investigated, PBPK models could help reconstruct and evaluate the exposure as part of the criminal evidence (Figure 3). Only a few papers were identified to describe PBPK models’ application in forensic science. To increase the use of these models a suggestion could be to provide a database of valid PBPK models, which forensic scientists could use to interpret the forensic evidence and to fill gaps, thereby informing a decision in hearings and trials. While such a database is beyond the scope of this review, a starting point could be the list of PBPK models developed for illicit drugs reported in the Supplementary Material. This list was extracted from the PBK model database [5].

As we have seen for ethanol (a well-studied substance of abuse), PBPK models can be used to predict blood alcohol concentrations directly from plasma or breath. Breath alcohol concentration could be used in alcohol intoxication situations, such as criminally driving under the influence (DUI) and related court proceedings. Additionally, while genetic variability is present in CYP2E1, ALDH, and ALD, the genetic profile for varying metabolism profiles has been and is being explored for these enzymes in order to better predict the clearance of ethanol in specific individuals and populations, which could be used in PBPK modeling. DDI drug-drug interactions as a result of alcohol inhibiting or inducing enzymes has also been explored. Furthermore, the accumulation of alcohol and its metabolites in organs, such as the brain and the liver, is important to consider when predicting possible PD outcomes. The combination of these detailed additions to a PBPK model would be crucial in forensic scenarios where one may need to recreate a drug interaction with ethanol to estimate over or underdosing (prodrug) in an individual or population. In this way, PBPK modeling can also be used to apply a reverse dosimetry approach, factoring in an individual’s metabolism status, to determine consumption of alcohol and possibly breath alcohol readings. The level of chronic alcohol use that would result in PD (clinical) outcomes of organ dysfunction or disease. While these scenarios of use have not been reported in forensic science presently, all are plausible uses of the PBPK modeling of ethanol (could also hold for other drugs of abuse); some may simply require a repurposing of currently available PBPK models, thereby reducing laboratory costs. However, uncertainties in alcohol concentration in PD outcomes and genetic variability exist and require more mechanistic research.

The value of PBPK modeling in environmental exposures for forensic purposes is evident, especially when considering life-stage modeling. Since PBPK modeling could use ontogeny equations to simulate many different life stages (infants, children, pregnant women, elderly, and organ impairment, etc.), it could be used to determine if toxic or detrimental internal concentrations were reached in these populations. PBPK modelling could impact future assessments and improve understanding of how the chemical(s) interact with the individual over a lifetime. Furthermore, if enough information becomes available, specific targets within a population could be modeled to estimate chemical sensitivities at specific sites, such as the brain or reproductive organs. Even if we have extensive information, there are always uncertainties for modelling research. To overcome these uncertainties, some techniques are applied for dealing with model uncertainties. Generally, model analysis, including uncertainty, sensitivity analysis and Bayesian calibration, and Monte Carlo population modelling are commonly used to reduce uncertainties and reflect variability. Uncertainty and sensitivity analysis help to identify the uncertain parameters with high impacts on model prediction. By applying Bayesian calibration, the uncertainties from parameter estimation and calibration can be reduced. In addition, the Monte Carlo population modelling, considering variabilities and distributions of model parameters, can help to simulate the PK profiles based on population uncertainties and variabilities.

The tissue concentrations of drugs and chemicals from forensic reports are valuable to validate the predictions from PBPK models and help to increase the confidence to apply PBPK models in humans. However, there are some limitations of data from forensic reports, which may even increase the uncertainties for modeling. First, compared to clinical studies, generally, forensic reports may not include all information, such as age, sex, ethnic factors, medical history, or the physical examination report, for individuals included in the forensic report. By improving this situation, more information should be included to reduce the uncertainties. Furthermore, PMR is also a factor with great impacts on applying tissue concentrations from autopsy samples for PBPK models. The PMR may lead to the differences in tissue concentrations of chemicals and drugs from post-mortem to ante-mortem samples. However, for chemicals with significant species differences in pharmacokinetics, such as PFOS, tissue concentrations from animal studies may not be good surrogates for human tissue concentrations. A recent study has compared the post-mortem to ante-mortem drug concentration ratios of 42 drugs [145]; thirty-five of those values are within 0.5–2, which are good enough to use for model validations; predictions of PBPK models within 2-fold differences from observed data are considered as good predictions. Application of computational models to predict RMP effects for chemicals and drugs would increase confidence in the use of tissue concentrations from post-mortem samples, especially for the chemicals with less RMP effects. By including tissue concentration data from forensic reports to validate model predictions, the studies of PBPK modeling should make a statement of inclusion or exclusion criteria and uncertainties for those tissue concentration data. Even though there are still some limitations to applying data from forensic reports for PBPK models, they are still invaluable data sources to validate the prediction of tissue concentrations from the PBPK models.

In conclusion, given the current uses of PBPK models in the pharmaceutical and regulatory fields, and their capability for prediction, we propose that they could provide a robust interpretation tool for forensic toxicologists, as previously mentioned by Bravo-Gomez et al. [20]. The potential applications of PBPK models could be significantly impactful for the interpretation of drug abuse and overdose data, as well as for environmental chemical accidents and adulteration.

**Table 1:** Drugs and chemicals with tissue concentrations from biopsy or autopsy samples.

Drug or Chemical Names	Cas Number	Tissues (Sample Type)	Sample Type (Sample Number)	Ethnicity	Sex	Age	References
**Alprazolam**	28981-97-7	Heart, subclavian blood, urine, bile, vitreous humor, liver, kidney	Autopsy	White	Female	44	Jenkins et al. 1997 [146]
**Amiodarone**	1951-25-3	Liver	Autopsy and biopsy	N/A	Both	17–78	Adams et al. 1985 [147]
**Amitriptyline**	549-18-8	Blood, pericardial fluid (PF), psoas muscle (PM), vitreous humor, vastus lateralis muscle (VM)	Autopsy (*n* = 9)	N/A	N/A	N/A	Åse Marit Leere Øiestad et al. 2018 [148]
**Amphetamine**	300-62-9	Blood, urine, liver	Autopsy	N/A	Male	30	Adjutantis et al. 1975 [149]
**Buprenorphine**	52485-79-7	Blood, urine, bile, liver, brain, kidney, myocardium, hair	Autopsy (*n* = 20)	N/A	Both	14–48	Tracqui et al. 1998 [150]
**Carbamazepine**	298-46-4	Blood, liver, kidney	Autopsy (*n* = 16)	N/A	Both	7–70	Klys et al. 2003 [151]
**Chloroquine**	54-05-07	Blood, liver, kidney, brain	Autopsy (*n* = 27)	N/A	Both	11 months–61	Di Maio et al. 1974 [155]
**Citalopram**	59729-33-8	Blood, bile, urine, liver, kidney	Autopsy (*n* = 13)	N/A	N/A	N/A	Levine et al. 2001 [156]
**Clozapine**	5786-21-0	Peripheral blood, heart blood, cerebrospinal fluid, vitreous humor, bile	Autopsy (*n* = 1)	N/A	Female	15	Keller et al. 1997 [157]
**Carfentanil**	59708-52-0	Blood, vitreous humor, urine	Autopsy (*n* = 290)	N/A	Both	16–69	Chatterton et al. 2020 [158]
**Codeine**	76-57-3	Blood, urine, bile, brain	Autopsy (*n* = 14)	N/A	Male	23–45	Gambaro et al. 2014 [159]
**Digoxin**	20830-75-5	Heart, skeletal muscle, liver, kidney	Autopsy	N/A	Both	Infants and children (2 days–7 years) Adult (59–80)	Kim et al. 1975 [160] Karjalainen et al. 1974 [161]
**Diltiazem**	42399-41-7	Blood, urine, bile	Autopsy (*n* = 1)	N/A	Female	78	Engelhart et al. 1997 [162]
**Diphenhydramine**	58-73-1	Blood (*n* = 44), liver (*n* = 33)	Autopsy	N/A	N/A	N/A	Levine et al.1996 [163]
**Fentanyl**	437-68-7	Central blood, femoral blood, brain, muscle, liver	Autopsy	N/A	Both	20–60	Chatterton et al. 2018 [164]
**Fluoxetine**	56296-78-7	Blood, urine, vitreous humor, bile, liver, lung, kidney, spleen, muscle, brain, heart	Autopsy (*n* = 10)	N/A	N/A	N/A	Johnson et al. 1992 [165]
**Lidocaine**	137-58-6	Blood, brain, heart, kidney, lung, spleen, skeletal muscle, adipose	Autopsy (*n* = 1)	N/A	N/A	64	Poklis et al. 1984 [166]
**Methadone**	76-99-3	Blood, bile, urine, liver, kidney, lung, brain	Autopsy (*n* = 6)	White, Black African	Both	18–46	Garriott et al. 1973 [167]
**Mirtazapine**	85650-52-8	Heart blood, peripheral blood, urine, liver, kidney, bile	Autopsy (*n* = 8)	N/A	Both	N/A	Moore et al. 1999 [168]
**Morphine**	57-27-2	Blood (*n* = 21), cerebrospinal fluid (*n* = 11), urine (*n* = 6)	Autopsy	N/A	Both	18–40 (1 unknown)	Bogusz et al. 1997 [169]
**Olanzapine**	132539-06-1	Peripheral blood, central blood, liver, vitreous humor	Autopsy (*n* = 28)	N/A	Both	26–68	Vance et al. 2009 [170]
**Oxycodone**	76-42-6	Blood, liver, urine, bile, vitreous humor	Autopsy (*n* = 36)	N/A	Both	21–62	Anderson et al. 2002 [171]
**PFOS**	Mixture (1763-23-1?)	Liver, kidney, adipose tissue, brain, basal ganglia, hypophysis, thyroid, gonads, pancreas, lung, skeletal muscle	Autopsy	N/A	Both	12–83	Maestri et al. 2006 [172]
**PFOA**	Mixture (335-67-1?)	Liver, kidney, adipose tissue, brain, basal ganglia, hypophysis, thyroid, gonads, pancreas, lung, skeletal muscle	Autopsy	NA	Both	12–83	Maestri et al. 2006 [172]
**PFAS**	mixture	Liver, Kidney, Lung, Brain, Bone (20 samples each)	Autopsy	Hispanic	N/A	28–83	Perez et al. 2013 [136]
**Quetiapine**	111974-69-7	Central blood, femoral blood, brain, muscle, liver	Autopsy	N/A	Both	20–60	Breivik et al. 2020 [173]
**THC**	1972-08-3	Blood, urine, liver, lung, kidney, spleen, muscle, brain, heart, bile	Autopsy	N/A	Male	22–69	Saenz et al. 2017 [174]
**Tramadol**	123154-38-1	Blood, urine, liver, kidney, bile	Autopsy (*n* = 8)	N/A	Both	28–67	Decker et al. 2007 [175]
**Venlafaxine**	93413-69-5	Blood, pericardial fluid (PF), psoas muscle (PM), vitreous humor, vastus lateralis muscle (VM)	Autopsy (*n* = 6)	N/A	N/A	N/A	Åse Marit Leere Øiestad et al. 2018 [151]
**Zolpidem**	82626-48-0	Blood, vitreous humor, bile, urine, gastric contents, liver	Autopsy (*n* = 2)	White, Black	Female	36, 58	Gock et al. 1999 [176]
**Zopiclone**	43200-80-2	Blood, liver	Autopsy (*n* = 2)	White, Indian	Both	24–82	Boniface et al. 1996 [177]

## Data Availability

Not applicable.

## Supplementary Materials

The following supporting information can be downloaded at: https://www.mdpi.com/article/10.3390/toxics11020126/s1, Table S1: Reporting a list of chemicals for which a PBPK model is available, reporting are the administration route, model compartments Simulation software used and reference/DOI.
