# Supplementary material for: An Overview of Physiologically-Based Pharmacokinetic Models for Forensic Science
Source: Toxics. 2023 Jan 28;11(2):126. doi: 10.3390/toxics11020126 (PMC9964742; doi:10.3390/toxics11020126)
Supplement: Supplementary file 1 [file toxics-11-00126-s001.zip › toxics-2019597-supplementary.pdf]

Supplementary Information to the manuscript by Fairman et al.

Table S1. Reporting a list of chemicals for which a PBPK model is available, reporting are the administration route, model compartments Simulation software used and reference/DOI. The information was extracted from the Thompson (2021, DOI: 10.1177/02611929211060264) database on PBPK models.

Chemical Name	Sex/ Life stage	Administration Route	Compartments or related information	Simulation Software	Reference	DOI
**alfentanil**	From birth	Intravenous infusion		Simcyp	Abduljalil et al, 2019, Clin Pharmacokinet, 59, 501-518	10.1007/s40262-019-00827-4
**alfentanil**		Intravenous bolus injection	adipose, brain, caecum, colon, duodenum, gut, heart, kidney, liver, lung, jejunum, ileum, marrow, muscle, reproductive organs, rest of body, skin, spleen	GastroPlus	Baneyx et al., 2014, Eur J Pharm Sci, 56, 1-15	10.1016/j.ejps.2014.02.002
**alfentanil**		Oral bolus	adipose, brain, caecum, colon, duodenum, gut, heart, kidney, liver, lung, jejunum, ileum, marrow, muscle, reproductive organs, rest of body, skin, spleen	GastroPlus	Baneyx et al., 2014, Eur J Pharm Sci, 56, 1-15	10.1016/j.ejps.2014.02.002

**buprenorphine**		Intravenous bolus injection	lung, adipose, bone, brain, heart, kidney, muscle, liver, portal vein, pancreas, spleen, gut, venous, arterial	Simcyp	Kalluri et al, 2017, Br J Clin Pharmacol, 83, 2458-2473	10.1111/bcp.13368
**buprenorphine**		Buccal	lung, adipose, bone, brain, heart, kidney, muscle, liver, portal vein, pancreas, spleen, gut, venous, arterial	Simcyp	Kalluri et al, 2017, Br J Clin Pharmacol, 83, 2458-2473	10.1111/bcp.13368
**buprenorphine**		Dermal injection	central compartment, periphery	Simcyp	Liu and Gobburu, 2018, J Pharm Sci, 107, 942-948	10.1016/j.xphs.2017.10.035
**buprenorphine**		Intravenous bolus injection	central compartment, periphery	Simcyp	Liu and Gobburu, 2018, J Pharm Sci, 107, 942-948	10.1016/j.xphs.2017.10.035
**carfentanil**	Male/adult	iv-administered		GastroPlus	Feasel et al. 2018	https://apps.dtic.mil/sti/pdfs/AD1060142.pdf
**cocaine**	Male	Intravenous bolus injection	lung, brain, fat, bone, heart, muscle, kidney, skin, spleen, intestine, liver, venous, arterial	MATLAB	Bravo-Gómez et al, 2019, Toxicol Res (Camb), 8, 432-446	10.1039/c8tx00309b
**cocaine**	Adult (typical)	Intravenous bolus injection		GastroPlus	Cheng et al, 2019, ACS Chem	10.1021/acschemneu

					Neurosci, 10, 3486-3499	ro.9b00109
**ethanol**		Inhalation	lung, fat, liver, brain, well/rapidly perfused, poorly/slowly perfused	acsIX	Crowell et al., 2015, Regul Toxicol Pharmacol, 73, 452-62	10.1016/j.yrtph.2015.07.021
**ethanol**		Metabolism from parent	lung, fat, liver, brain, well/rapidly perfused, poorly/slowly perfused	acsIX	Crowell et al., 2015, Regul Toxicol Pharmacol, 73, 452-62	10.1016/j.yrtph.2015.07.021
**ethanol**		Inhalation	bone marrow, fat, gastrointestinal tract, kidney, liver, lung, poorly/slowly perfused, well/rapidly perfused		Dennison, Bigelow and Andersen, 2004, Toxicol Ind Health, 20, 165-75	10.1191/0748233704th203oa
**ethanol**		Oral bolus	bone marrow, fat, gastrointestinal tract, kidney, liver, lung, poorly/slowly perfused, well/rapidly perfused		Dennison, Bigelow and Andersen, 2004, Toxicol Ind Health, 20, 165-75	10.1191/0748233704th203oa
**ethanol**	Male/Adult	Inhalation	brain, fat, liver, lung, poorly/slowly perfused, well/rapidly perfused	acsIX	Dumas-Campagna et al., 2014, Inhal Toxicol, 26, 59-69	10.3109/08958378.2013.853714

**ethanol**	Female/adult	Inhalation	brain, fat, liver, lung, poorly/slowly perfused, well/rapidly perfused	acsIX	Dumas-Campagna et al., 2014, Inhal Toxicol, 26, 59-69	10.3109/08958378.2013.853714
**Ethanol**		Intravenous bolus injection		PKQuest	Levitt, 2002, BMC Clin Pharmacol, 2, 4	10.1186/1472-6904-2-4
**ethanol**		Oral bolus		PKQuest	Levitt, 2002, BMC Clin Pharmacol, 2, 4	10.1186/1472-6904-2-4
**ethanol**		Oral bolus	gut, liver, lung, muscle, stomach, well/rapidly perfused, poorly/slowly perfused	MATLAB	MacDonald et al, 2002, Regul Toxicol Pharmacol, 35, 165-76	10.1006/rtph.2001.1507
**ethanol**	Male/From birth	Inhalation	brain, fat, liver, lung, poorly/slowly perfused, well/rapidly perfused	Multiple software used	Pastino et al., 1997, Toxicol Appl Pharmacol, 145, 147-57	10.1006/taap.1997.8161
**ethanol**	Male/From birth	Intravenous infusion	brain, fat, liver, lung, poorly/slowly perfused, well/rapidly perfused	Multiple software used	Pastino et al., 1997, Toxicol Appl Pharmacol, 145, 147-57	10.1006/taap.1997.8161
**ethanol**	Male (Caucasian)	Inhalation	brain, fat, gastrointestinal tract, liver, lung, poorly/slowly		Pastino, Flynn and Sultatos, 2000, Drug Chem Toxicol, 23, 179-201	10.1081/dct-100100110

			perfused, well/rapidly perfused			
**ethanol**	Male (African American)	Inhalation	brain, fat, gastrointestinal tract, liver, lung, poorly/slowly perfused, well/rapidly perfused		Pastino, Flynn and Sultatos, 2000, Drug Chem Toxicol, 23, 179-201	10.1081/dct-100100110
**ethanol**	Male (Caucasian)	Oral bolus	brain, fat, gastrointestinal tract, liver, lung, poorly/slowly perfused, well/rapidly perfused		Pastino, Flynn and Sultatos, 2000, Drug Chem Toxicol, 23, 179-201	10.1081/dct-100100110
**ethanol**	Male (African American)	Oral bolus	brain, fat, gastrointestinal tract, liver, lung, poorly/slowly perfused, well/rapidly perfused		Pastino, Flynn and Sultatos, 2000, Drug Chem Toxicol, 23, 179-201	10.1081/dct-100100110
**ethanol**		Inhalation			Plawecki et al., 2007, Biomed Signal Process Control, 2, 97-110	10.1016/j.bspc.2007.04.001
**ethanol**	Male/Adult	Intravenous infusion			Ramchandani et al., 2009, Alcohol Clin Exp Res, 33, 938-44	10.1111/j.1530-

						0277.2009.00906.x
**ethanol**	Female/adult	Intravenous infusion			Ramchandani et al., 2009, Alcohol Clin Exp Res, 33, 938-44	10.1111/j.1530-0277.2009.00906.x
**fentanyl**		Intravenous infusion	arterial, brain, carcass, fat, gut, heart, kidney, liver, lung, muscle, pancreas, skin, spleen, venous	MATLAB	Björkman, 2003, J Pharmacokinet Pharmacodyn, 30, 285-307	10.1023/a:1026194618660
**fentanyl**	Adult (typical)	Intravenous bolus injection		GastroPlus	Cheng et al, 2019, ACS Chem Neurosci, 10, 3486-3499	10.1021/acschemneuro.9b00109
**fentanyl**		Intravenous bolus injection		MATLAB	Huang and Isoherranen, 2019, J Pharmacol Exp Ther, 372, 30-45	10.1124/jpet.119.262154
**fentanyl**		Buccal		MATLAB	Huang and Isoherranen, 2019, J Pharmacol Exp Ther, 372, 30-45	10.1124/jpet.119.262154
**methadone**	Female/pregnant	Oral bolus		Multiple software used	Ke et al., 2014, Br J Clin Pharmacol, 77, 554-70	10.1111/bcp.12207
**methadone**		Intravenous bolus injection	adipose, bone, brain, gastrointestinal tract, heart, kidney, liver, lung, marrow,	MATLAB	Yang et al., 2006, J Pharmacokinet Pharmacodyn, 33, 485-518	10.1007/s10928-006-9018-0

			muscle, pancreas, skin, spleen, thyroid			
**methadone**		Oral bolus	adipose, bone, brain, gastrointestinal tract, heart, kidney, liver, lung, marrow, muscle, pancreas, skin, spleen, thyroid	MATLAB	Yang et al., 2006, J Pharmacokinet Pharmacodyn, 33, 485-518	10.1007/s10928-006-9018-0
**morphine**	Adult (typical)	Intravenous bolus injection		Simcyp	Emoto et al, 2017, CPT Pharmacometrics Syst Pharmaacol, 6, 110-119	10.1002/psp4.12144
**morphine**	From birth	Intravenous bolus injection		Simcyp	Emoto et al, 2017, CPT Pharmacometrics Syst Pharmaacol, 6, 110-119	10.1002/psp4.12144
**morphine**	From birth	Intravenous bolus injection		Simcyp	Emoto et al, 2018, CPT Pharmacometrics Syst Pharmacol, 7, 464-473	10.1002/psp4.12306
**morphine**		Intravenous bolus injection	lung, brain, muscle, adipose, marrow, intestine, spleen, liver, kidney, arterial, venous		German, Pilvankar and Przekwas, 2019, J Pharmacokinet Pharmacodyn, 46, 513-529	10.1007/s10928-019-09648-1
**morphine**		Intravenous infusion	lung, brain, muscle, adipose, marrow, intestine, spleen,		German, Pilvankar and Przekwas, 2019, J Pharmacokinet	10.1007/s10928-019-09648-1

			liver, kidney, arterial, venous		Pharmacodyn, 46, 513-529	
**morphine**		Intravenous bolus injection	liver	Simcyp	Prasad et al., 2018, Drug Metab Dispos, 46, 943-952	10.1124/dmd.118.080523
**morphine**	From birth	Intravenous bolus injection	liver	Simcyp	Prasad et al., 2018, Drug Metab Dispos, 46, 943-952	10.1124/dmd.118.080523
**morphine**	Male/Adult	Intravenous bolus injection		PK-Sim/MoBi	Schlender et al, 2016, Clin Pharmcokinet, 55, 1573-1589	10.1007/s40262-016-0422-3
**morphine**	Female/adult	Intravenous bolus injection		PK-Sim/MoBi	Schlender et al, 2016, Clin Pharmcokinet, 55, 1573-1589	10.1007/s40262-016-0422-3
**morphine**	Male/old age	Intravenous bolus injection		PK-Sim/MoBi	Schlender et al, 2016, Clin Pharmcokinet, 55, 1573-1589	10.1007/s40262-016-0422-3
**morphine**	Female/old age	Intravenous bolus injection		PK-Sim/MoBi	Schlender et al, 2016, Clin Pharmcokinet, 55, 1573-1589	10.1007/s40262-016-0422-3
**morphine**	From birth	Intravenous infusion		PK-Sim/MoBi	Strougo et al., 2012, J Pharmacokinet Pharmacodyn, 39, 195-203	10.1007/s10928-012-9241-9
**morphine**	Adult (typical)	Intravenous infusion	plasma, brain	NONMEM	Yamamoto et al, 2018, Eur J Pharm Sci, 112, 168-179	10.1016/j.ejps.2017.11.011
**N,O-didesmethyltramadol**	Adult (typical)	Intravenous bolus injection		Simcyp	T'jollyn et al., 2015, Pharm Res, 32, 260-74	10.1007/s11095-014-1460-x

**N-desmethyltramadol**	Adult (typical)	Metabolism from parent		Simcyp	T'jollyn et al., 2015, Pharm Res, 32, 260-74	10.1007/s11095-014-1460-x
**O-desmethyltramadol**	Adult (typical)	Metabolism from parent		Simcyp	T'jollyn et al., 2015, Pharm Res, 32, 260-74	10.1007/s11095-014-1460-x
**oxycodone**	Adult (typical)	Intravenous infusion	plasma, brain	NONMEM	Yamamoto et al, 2018, Eur J Pharm Sci, 112, 168-179	10.1016/j.ejps.2017.11.011
**oxycodone**	From birth	Intravenous bolus injection		PK-Sim/MoBi	Zheng et al., 2019, Pharm Res, 36, 171	10.1007/s11095-019-2708-2
**oxycodone**	From birth	Oral bolus		PK-Sim/MoBi	Zheng et al., 2019, Pharm Res, 36, 171	10.1007/s11095-019-2708-2
**oxycodone**	Male/Adult	Intravenous bolus injection		PK-Sim/MoBi	Zheng et al., 2019, Pharm Res, 36, 171	10.1007/s11095-019-2708-2
**oxycodone**	Male/Adult	Oral bolus		PK-Sim/MoBi	Zheng et al., 2019, Pharm Res, 36, 171	10.1007/s11095-019-2708-2
**remifentanil**		Intravenous infusion	plasma, tissue, gastrointestinal tract small intestine, large intestine, gall bladder, liver, poorly/slowly perfused, well/rapidly perfused		Abbiati and Manca, 2017, Curr Drug Deliv, 14, 190-202	10.2174/1567201813666160524142031

**remifentanil**		Intravenous infusion	large intestine, small intestine, stomach, liver, plasma, poorly/slowly perfused, well/rapidly perfused	MATLAB	Cascone et al, 2018, Eur J Phar Sci, 111, 20-28	10.1016/j.ejps.2017.09.028
**remifentanil**		Intravenous bolus injection	large intestine, small intestine, stomach, liver, plasma, poorly/slowly perfused, well/rapidly perfused	MATLAB	Cascone et al, 2018, Eur J Phar Sci, 111, 20-28	10.1016/j.ejps.2017.09.028
**R-methadone**	Female/pregnant	Oral bolus		Multiple software used	Ke et al., 2014, Br J Clin Pharmacol, 77, 554-70	10.1111/bcp.12207
**S-methadone**	Female/pregnant	Oral bolus		Multiple software used	Ke et al., 2014, Br J Clin Pharmacol, 77, 554-70	10.1111/bcp.12207
**sufentanil**	Children	Intravenous bolus injection		SimCyp	Zhou et al., 2018	https://doi.org/10.1002/cpt.904
**sufentanil**	Adult (typical)	Intravenous bolus injection		SimCyp	Zhou et al., 2018	https://doi.org/10.1002/cpt.905
**tramadol**	Adult (typical)	Intravenous bolus injection		Simcyp	T'Jollyn et al, 2015, AAPS J, 17, 1376-87	10.1208/s12248-015-9803-z

**tramadol**	Adult (typical)	Intravenous bolus injection		Simcyp	T'jollyn et al., 2015, Pharm Res, 32, 260-74	10.1007/s11095-014-1460-x
